# Poly-ε-Caprolactone 3D-Printed Porous Scaffold in a Femoral Condyle Defect Model Induces Early Osteo-Regeneration

**DOI:** 10.3390/polym16010066

**Published:** 2023-12-24

**Authors:** Arianna De Mori, Aikaterina Karali, Evangelos Daskalakis, Richard Hing, Paulo Jorge Da Silva Bartolo, Glen Cooper, Gordon Blunn

**Affiliations:** 1 School of Pharmacy and Biomedical Sciences, University of Portsmouth, St. Michael’s Building, White Swan Road, Portsmouth PO1 2DT, UK; 2 Zeiss Global Centre, School of Mechanical and Design Engineering, University of Portsmouth, Portsmouth PO1 3DJ, UK; 3 School of Mechanical, Aerospace and Civil Engineering, University of Manchester, Manchester M13 9PL, UKglen.cooper@manchester.ac.uk (G.C.); 4 School of Earth and Environmental Sciences, University of Portsmouth, Portsmouth PO1 2HB, UK

**Keywords:** polycaprolactone, hydroxyapatite, Bioglass, beta-tricalcium phosphate, three-dimensional printing, bone tissue engineering, in vivo

## Abstract

Large bone reconstruction following trauma poses significant challenges for reconstructive surgeons, leading to a healthcare burden for health systems, long-term pain for patients, and complex disorders such as infections that are difficult to resolve. The use of bone substitutes is suboptimal for substantial bone loss, as they induce localized atrophy and are generally weak, and unable to support load. A combination of strong polycaprolactone (PCL)-based scaffolds, with an average channel size of 330 µm, enriched with 20% *w*/*w* of hydroxyapatite (HA), β-tricalcium phosphate (TCP), or Bioglass 45S5 (Bioglass), has been developed and tested for bone regeneration in a critical-size ovine femoral condyle defect model. After 6 weeks, tissue ingrowth was analyzed using X-ray computed tomography (XCT), Backscattered Electron Microscopy (BSE), and histomorphometry. At this point, all materials promoted new bone formation. Histological analysis showed no statistical difference among the different biomaterials (*p* > 0.05), but PCL-Bioglass scaffolds enhanced bone formation in the center of the scaffold more than the other types of materials. These materials show potential to promote bone regeneration in critical-sized defects on load-bearing sites.

## 1. Introduction

Large bone reconstruction following trauma poses significant challenges for reconstructive surgeons and results in a healthcare burden and long-term pain for patients [1,2]. High-impact traumas can lead to complex disorders (e.g., infection, inadequate perfusion, and devitalized tissues), that are difficult to resolve and often end with amputation [3,4]. Autologous bone grafting remains the gold standard in bone repair [5]; however, it is associated with clinical setbacks, such as the limited availability of healthy bone, high costs, and morbidity at the bone harvesting site. Alternative treatments, such as the use of external fixators and plates, have several drawbacks, including high risk of infection, chronic pain, need for revision surgery, time-consuming and costly treatments, and increased morbidity [6,7]. Conversely, the use of bone substitutes induces localized atrophy when a large volume is used (over 60 cm^3^), and their vascularization remains suboptimal [8]. Given the patient-specific nature of bone lesions, artificial bone grafts must be meticulously customized to address this challenge [9]. Biomaterials employed in the reconstruction of bone tissue require implants to have properties which include interconnected pores (ranging from 100 to 500 µm), facilitating cell migration, bioactivity, biodegradability, biocompatibility, and cost-effectiveness [10,11,12,13]. Considering all these challenges, new engineering techniques, such as computer-aided additive manufacturing (e.g., 3D printing), may offer a solution for the design of tailored scaffolds of a specific shape, size, mechanical performance, and porosity [14,15]. In this context, extrusion-based techniques have the advantage of avoiding the use of organic solvents which can be cytotoxic [16,17].

Poly-ε-caprolactone (PCL) is a thermoplastic used in 3D printing that has been extensively investigated for use in bone scaffolds due to its toughness and ability to integrate with tissues [18]. Nevertheless, PCL is a hydrophobic material that does not promote initial cell attachment [19]. Additionally, it degrades very slowly in vivo, reducing the speed of tissue replacement [20]. Consequently, researchers have used different strategies to improve PCL’s osteoconductivity, degradation rate and strength, such as the inclusion of bioactive ceramics (e.g., calcium-phosphate based materials and bioglasses) [17]. One of the first studied bioceramics was hydroxyapatite (HA) thatis the natural mineral component of bone, with the formula Ca_5_(PO_4_)_3_OH. HA is stable at physiological conditions, is biocompatible, and is bioactive, promoting the formation of apatite on its surface. However, HA is brittle, has low mechanical stability, shows limited or no osteoinductivity, and only slowly degrades in the body [14,17,21]. An alternative option is β-tricalcium phosphate (β-TCP) (Ca_3_(PO_4_)_2_), which has excellent biocompatibility, osteoconductivity, and faster biodegradation. Bioglass is another bone graft substitute, and in contrast to calcium phosphate materials such as HA and β-TCP contains a combination of oxides, including silica (SiO_2_), sodium oxide (CaO), and phosphorus pentoxide (P_2_O_5_) [22]. This combination imparts bioactivity, triggers blood vessel formation, and releases ions (silica, calcium, sodium, and phosphate) that stimulate osteogenesis and angiogenesis [23,24,25]. Yuan et al. demonstrated the osteoinductive properties of Bioglass by employing porous foam implants in the thigh muscles of dogs, following a 3-month in vivo experimental period [26]. Furthermore, an in vivo study by Algarni et al. revealed that bioactive glasses have no harmful inflammatory effects, and there is no foreign body reaction [27].

In vitro and in vivo comparative studies of these three ceramics materials are quite limited, and not conclusive. For example, Ghosh et al. tested different bone struts made of pure hydroxyapatite, β-TCP, and Bioglass in the radius bone of Bengal goats [28]. They found that, 90 days after surgery, lower bone ingrowth and reduced strength were observed with HA compared to β-TCP and Bioglass-based implants. Zin et al. compared different bioceramic surfaces in rabbit muscle, highlighting that β-TCP did not promote CaP formation in vivo, when compared with HA, α-TCP, and Bioglass. Przybilla et al. tested HA, β-TCP, and Bioglass as a 20 µm thin ceramic coating of titanium implants in vivo (in a rabbit model for up to 24 weeks). Their results demonstrated that all the coatings promoted strong implant fixation and high bone contact, except for Bioglass, and they suggested that Bioglass resorbed faster than the formation of new bone tissue [29]. Razavi et al. compared the in vivo bone regeneration (up to 45 days) of nano-HA and nano-BG scaffolds implanted on canine crestal ridges. Overall, nano-HA demonstrated higher bone regeneration (especially in the first 30 days) compared to nano-BG [30]. Poh et al. manufactured PCL scaffolds containing either 50% Bioglass, strontium-substituted bioactive glass (SrBG), or calcium phosphate (CaP) and tested their ability to support osteogenesis in vitro, and osteoinductivity in vivo [31]. While, in vitro, the PCL-CaP, PCL-SrGB, and PCL-Bioglass promoted the upregulation of osteopontin and osteocalcin, no bone formation was observed in any scaffold group, for both 8 and 16 weeks, indicating their inability to promote osteoinductivity. Helaehil et al. investigated the use of polymeric PCL/β-TCP (20 wt%) and PCL/HA (20 wt%) scaffolds (pore size 350 µm), both in vitro and in vivo. In vitro, PCL/HA scaffolds showed greater biocompatibility and cell proliferation than PCL/β-TCP scaffolds, whilst PCL/β-TCP scaffolds had better mechanical properties than PCL/HA scaffolds. After implantation for 30, 60, and 120 days (in Wistar rats bone defects), no significant difference was seen in mineralization when PCL, PCL/β-TCP and PCL/HA were compared [32].

In a previous study, we investigated the potential of anatomically designed scaffolds (referred to as “bone bricks’’) made of PCL and different ceramics (hydroxyapatite, tri-calcium phosphate, and Bioglass) to support the osteogenesis of hAdMSC in vitro [33]. Our findings indicated that bone bricks containing PCL/TCP had the best mechanical performance and PCL/HA promoted the highest cell proliferation, whereas cells on PCL/Bioglass showed the highest production of osteogenic differentiation markers (alkaline phosphatase and osteocalcin). Further rheological and mechanical studies confirmed the blends’ suitability for manufacturing the scaffolds, and their use in load-bearing applications [33,34,35].

In our present study, we implanted porous 3D PCL scaffolds, designed with 20 wt% of Bioglass, HA, or TCP in a critical-size sheep femoral condyle defect. The main goal of this study was to compare, for the first time, the in vivo osteointegration and osteoconductive effectiveness of 3D-printed scaffolds made of PCL, PCL/HA, PCL/Bioglass and PCL/TCP. After a period of 6 weeks, tissues adjacent to the implants and within the porous structure were analyzed by means of X-ray computed tomography (XCT), Backscattered Electron Microscopy (BSE) and histomorphometry.

## 2. Materials and Method

### 2.1. Materials

Basic fuchsin, β-tricalcium phosphate (TCP) (<10 μm particles size, Mw = 310.18 r/mol, MP = 1391 °C), DPX mountant for histology, hydrogen peroxide 30%, paraformaldehyde, sodium chloride, sodium tetraborate, and toluidine blue O were purchased from Sigma-Adrich (Gillingham, UK). Ethanol (100%) and acetic acid (99%) were bought from Fisher (Loughborough, UK). LR White Resin (Hard) and accelerator for London resin were purchased from Agar Scientific (Stansted, UK). Bioglass 45S5 (Bioglass) (<10 µm), with a composition of 45 wt% SiO_2_, 24.5 wt% CaO, 24.5 wt% Na_2_O, and 6 wt% P_2_O_5_, and hydroxyapatite nanopowder (<20 nm particles size, Mw = 502.31 r/mol, MP = 1100 °C) were supplied by Cera Dynamics Ltd. James Kent Group (Stoke, UK) in powder form. PCL (CAPA 6500, molecular weight = 50,000 Da, melting point = 60 °C) was purchased from Perstorp (Warrington, UK).

### 2.2. Fabrication of PCL Plugs

**Figure 1:**
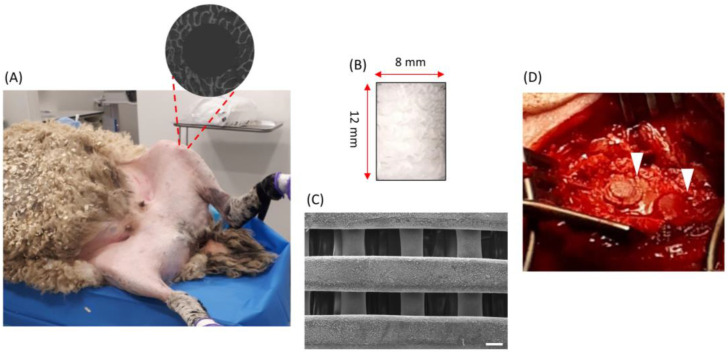
(**A**) Photo taken during the surgical procedure. In total, each sheep had four defects: two in each distal-medial femoral condyle of both the right and left hind limbs. The grey circle is a schematic representation of a femoral condyle defect which was obtained through the drilling process. (**B**) The scaffolds had a cylindrical shape with a length of 12 mm and a dimeter of 8 mm. (**C**) SEM image of the top-section of one of the scaffolds. The scale bar is 200 µm. (**D**) Image of plugs (indicated by white arrows) implanted in the condyle defects.

PCL was blended by melting the polymer and mixing it with different bioceramics: 20 wt% of HA or β-TCP, or Bioglass. Briefly, PCL pellets were melted at 150 °C in a porcelain bowl using a hot plate and homogenous heating was achieved via manual stirring (due to the thickness of the blend). Composite materials were mixed for around 1 h to ensure a uniform distribution of the ceramics in the polymer matrix. Four different plugs were fabricated: PCL, PCL containing 20 wt% of HA, PCL containing 20 wt% of β-TCP, and PCL containing 20 wt% of Bioglass. Porous scaffolds were produced in the form of square blocks (30 × 30 × 30 mm) using a screw-assisted extrusion-based additive manufacturing machine (3D Discovery, RegenHU, Villaz-Saint-Pierre, Switzerland). The parameters used for the scaffold production are as follows: printing temperature of 90 °C, deposition velocity of 20 mm/s, screw rotation velocity of 6 rpm, and a metallic needle with an inner diameter of 330 μm. Then, the scaffolds were cut into round plugs with the use of a metallic pump to obtain the desirable diameter (8 mm). For animal studies, the plugs were sterilized in 80% EtOH for 24 h, before being rinsed with phosphate-buffered saline and dried for 24 h. Finally, the plugs were exposed to UV light for 20 min. The plugs had a homogeneous cuboidal pore size of 330 μm, as shown in Figure 1C.

### 2.3. Surgical Procedure

The procedure was reviewed by the Animal Welfare and Ethical Review Board at the Royal Veterinary College, London, UK. It was conducted in accordance with the UK Home Office License No P16F4AA0A, complying with the Animal (Scientific Procedures) Act. Six adult female sheep (weight > 70 kg) were enrolled in this study. The animals were sedated by intravenous administration of ketamine and midazolam, with sedation maintained using gaseous anesthesia with 2.5% isoflurane, administered via an endotracheal tube [36]. Each animal had 4 implants inserted into four 8 mm × 12 mm drilled defects (Figure 1B), two in each distal medial femoral condyle of the right and left hind limbs. Every animal received four different scaffolds (*n* = 6) (Figure 1A,D). The position of each implant within the four sites was rotated. Previous studies assessing bone ingrowth utilized a similar model with comparable implant sizes [37]. An empty defect was not considered necessary since previous findings established the defect to be of a critical size. Moreover, one of the PCL implants in each animal was not filled with any bioactive materials. Finally, to utilize the least number of animals it was decided not to perform an empty defect. The decision to use an N of 6 was based on a power calculation using data from previous experiments and using a clinically relevant difference between control and experimental implants. Immediately prior to surgery, animals received an intramuscular dose of antibiotics that was repeated daily for two days post-surgery. Pain relief was provided by Fentanyl transdermal patches (Janssen-Cilag Ltd., High Wycombe, UK) that were changed 2 days after surgery, and discontinued 4 days post-surgery. Following surgery, animals were housed in individual pens, allowed to bear weight, and were given food, and water ad libitum. After two weeks, they were fully weight-bearing and group-housed in a large indoor barn. All animals were euthanized 6 weeks after surgery using an overdose of sodium pentobarbitone. The implants, surrounded by bone, were removed, and analyzed as detailed below.

### 2.4. X-ray Computed Tomography Imaging

X-ray Computed tomography (XCT) imaging was performed using a Versa 520 system (Carl Zeiss Microscopy, White Plains, NY, USA). Saline solution was used to keep the condyle bone specimens hydrated throughout the duration of the imaging. The system operated at 70 kV/6 W with exposure time set at 2 s/projection (1601 projections); using a 0.4× objective, resulting in approximately 25 µm isotropic voxel size. The resulting field of view (FOV) included the bone bricks and the surrounding mature trabecular tissue.

### 2.5. Image Post-Processing

The 3D datasets obtained from the XCT imaging were reconstructed with no ring removal and the rotation angle removal and the rotation angle shift was specimen specific as computed by the integrated software (Scout-and-Scan Control System Reconstruct v. 16.0 (Carl Zeiss Microscopy, Pleasanton, CA, USA) and each dataset consisted of 1018 images (1002 *×* 1024 pixels, 16-bit grey levels). The datasets were then processed using Avizo 9.7 (Thermo Fisher Scientific, Waltham, MA, USA) software. The region of interest (ROI) was identified by the location of the implant.A cylindrical mask with a diameter of 10 mm, consisting of the 8 mm diameter implant and 1 mm of surrounding tissue was created covering the ROI. The newly formed bone and bone bricks were segmented separately, using an interactive thresholding and a volumetric analysis was executed on the ROI to identify the percentage of newly formed bone (bone volume/total ROI volume). The range for interactive thresholding was 2500–3000 Hounsfield Units.

### 2.6. Histology

After the XCT imaging, the bone specimens were fixed in 4% paraformaldehyde, at 4 °C, for a minimum of two days. They were then rinsed in saline solution (0.9% NaCl) for 2 h, before undergoing dehydration in increasing concentrations of ethanol (from 50% to 100%). The specimens were kept in 100% ethanol for 5 days, with the solution changed daily. Defatting the tissues with chloroform was avoided because PCL is soluble in this solvent. Subsequently, the specimens were infiltrated in LR White Resin (Agar Scientific, Stansted, UK), at 4 °C by submersion in non-polymerized resin (in 60 mL PP containers) (Fisher, Loughborough, UK) for 48 h, with the resin changed twice. Vacuum was used to ensure proper infiltration of the resin within the specimens. The used resin was then replaced with 40 mL of fresh resin and polymerization was carried out after addition of the accelerator (Agar Scientific, Stansted, UK), according to the manufacturer’s instructions, at 4 °C for 20 min. The embedded materials were sectioned in the transverse plane (perpendicular to the implants long axis) using a diamond-coated saw blade mounted on a Leica SP1600 saw microtome (Numloch, Germany) to obtain semi-thin sections with a thickness of approximately 100 μm. The sections were then mounted on ultraviolet transmitting acrylic slides (Agar Scientific, Stansted, UK) with Loctite Super Glue Precision Max (Winsford, UK). Subsequently, the specimens were sanded and polished with 1200, 2500 and 4000 grade silicon carbide paper (Struers Ltd., Rotherham, UK). Afterwards, the polished sections were etched in a bath of 30% hydrogen peroxide, for 5 min. Then, the sections were rinsed twice under tap water and placed in a 10% acetic acid bath (1 min) and then rinsed again. A 1% toluidine Blue O solution with sodium tetraborate was used, and a toluidine/basic fuchsin solution (730 mg of toluidine blue O and 270 mg of basic fuchsin in 100 mL of 30% ethanol) was added for counterstaining [38,39]. Toluidine blue was used to stain soft tissues blue, whilst fuchsin stained the bone tissue pink. The sections were finally mounted with DPX Mountant for histology (Merck Life Science, Gillingham, UK). Then sections were imaged using an optical microscope (GXM-L1500BHTG, GTVision, Sudbury, UK). Histomorphometry analyses were conducted on the images, which included evaluating the percentage of new bone formation, soft tissue formation, and residual scaffolds. The quantities of the different tissues (bone, soft tissue, and scaffold), as well as the points of contact between bone-scaffold and soft tissue-scaffold were assessed using a line intercept analysis. This analysis was performed by superimposing a 10 × 10 square grid onto each examined image and where these lines intersected the presence of soft tissue, bone or scaffold was recorded enabling the level of osteointegration to be assessed.

### 2.7. Backscattered Electron Microscopy

Unstained polished sections of the resin-embedded implants were imaged using backscattered scanning electron microscopy (SEM-EVO MA10, Zeiss, White Plains, NY, USA) equipped with Oxford Aztec (Zeiss, USA). To increase the conductivity of the materials and, consequently, image quality, copper SEM tape was attached to each sample and to the stub prior to the imaging and samples were sputter coated with a layer of gold palladium (Polaron e500) (Quorum Technologies, Laughton, UK). SEM parameters chosen for imaging were electron high tension (EHT) 20.00 kV, magnification 26–30× and, I probe 600 pas. Images were analyzed for new bone formation (%) using ImageJ v. 1.8.0 (NIH, Madison, WI, USA). A circular region (ROI), corresponding to the defect area, was initially cropped. Then, both the total area of the defect and the area of the new bone were binarized using an interactive threshold method (default). Thresholding enabled the isolation and quantification of bone formation based on greyscale. The percentage of new bone formation was determined according to the following formula:

(1) $$\left(\frac{A\;bone}{A\;tot}\right)\times 100$$

where, *A bone* represents the area (in pixels) of the new bone ingrowth and *A tot* is the total area of interest.

### 2.8. Statistical Analysis

Data are expressed as the mean ± standard deviation (SD). Normality was assessed with a Shapiro–Wilk normality test (level of significance = 0.05), and a comparison between groups made as indicated in the caption of each figure. The statistical analysis was performed with GraphPad Prism 9 (La Jolla, CA, USA). Data were considered significant when *p* < 0.05.

## 3. Results

### 3.1. Histomorphometric Analysis

**Figure 2:**
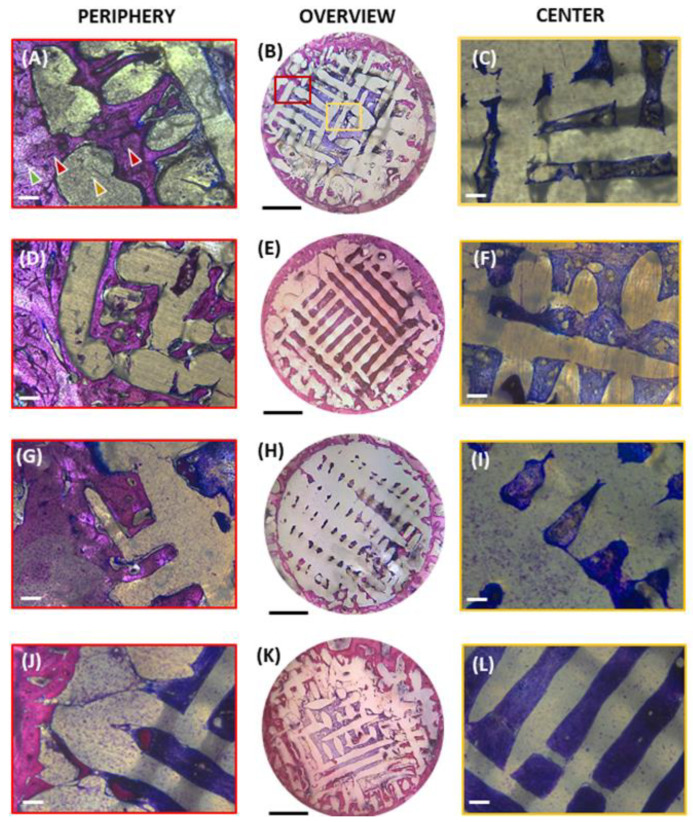
Representative fuchsin/toluidine blue-stained images of cross-sections of different scaffold six weeks after implantation. The first row represents PCL, second row PCL-HA, third row PCL-TCP, and the fourth row PCL-Bioglass. An overview of the samples is provided in the image labelled with (**B**,**E**,**H**,**K**), corresponding to PCL, PCL-HA, PCL-TCP, and PCL-Bioglass, respectively. The scale bar is set to = 2 mm. In (**B**), the red and yellow delineated regions represent the periphery and center of defect, respectively. Subsequently, these regions were photographed at higher magnifications for quantitative analysis of tissue formation. PCL-based plugs appear white as they do not retain any staining, while the connective tissue is stained blue, and the mineralized bone matrix appears pink. Representative areas at the periphery of the defects are labelled: PCL (**A**), PCL-HA (**D**), PCL-TCP (**G**), and PCL-Bioglass (**J**). Representative areas at the center of the defects are labelled: PCL (**C**), PCL-HA (**F**), PCL-TCP (**I**), and PCL-Bioglass (**L**). The scale bar is set to = 250 µm. The samples show portions of pre-existing bone stained in a lighter purple color (and indicated by a green arrow). Within the defects, bone bricks remain intact (shown by the yellow arrow), while soft tissue (pointed out by the grey arrow) had filled all the empty spaces within the biomaterial walls. New bone formation (indicated by red arrows) is highlighted by a darker purple color, filling the spaces previously occupied by soft tissue.

**Figure 3:**
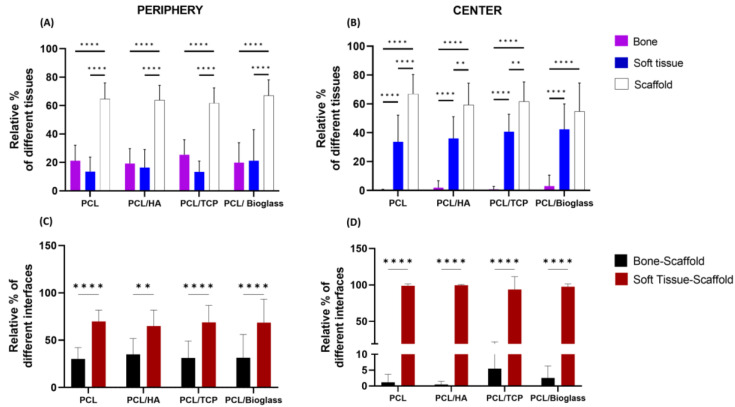
Graphs showing new bone formation and the frequency of different interfaces. The percentage of new bone formation (in purple), soft tissue (in blue) and residual biomaterial (in white) is shown for both the periphery (**A**) and in the center of the bone defect (**B**). Graphs representing interphases/cm^2^ in the periphery (**C**) and center (**D**) of the bone defect are presented. The results are presented as mean ± SD (*n* ≥ 9). Two-way ANOVA was performed among different groups. ** *p* < 0.01, and, **** *p* < 0.0001 were used to denote significance levels.

Histological staining of undecalcified sections provides insights into tissue regeneration within the porous structure and compatibility of the materials. In our study, we analyzed two regions of interest (ROI): the center and the periphery of the bone defects (as shown in Figure 2B,E,H,K). Examples of images analyzed at the periphery of the defects (red framed) are shown in: Figure 2A,D,G,J for PCL, PCL-HA, PCL-TCP, and PCL-Bioglass, respectively. Examples of images analyzed at the center of the defects (yellow framed) are shown in: Figure 2C,F,I,L for PCL, PCL-HA, PCL-TCP, and PCL-Bioglass, respectively. Subsequently, we calculated the relative percentage of each tissue type (Figure 3A,B) and of the different interfaces (Figure 3C,D).

As shown in Figure 2, staining revealed that all the defects exhibited the formation of new bone and fibrous tissue within the interconnected pore network of the biomaterials. Furthermore, all the defects were surrounded by trabecular bone (light pink) that had become more sclerotic, which was integrated with the periphery of the scaffold. Over 50% of the defect region was filled with scaffold, both in the center and periphery of the defects (Figure 3A,B). In particular, the percentage of area occupied by the scaffold at the center was as follows: 67.61 ± 13.70%, 59.30 ± 15.08%, 61.71 ± 13.51%, and 54.77 ± 19.67% for PCL, PCL-HA, PCL-TCP, and PCL-Bioglass, respectively (*p* > 0.05). At the periphery, the amounts were: 64.7 ± 11.27%, 63.89 ± 10.41%, 61.73 ± 10.68%, and 67.07 ± 11.07% for PCL, PCL-HA, PCL-TCP, and PCL-Bioglass, respectively (*p* > 0.05). The newly formed bone that stained a darker pink than mature bone developed at the defect’s periphery, while the central core was predominantly occupied by fibrous connective tissue (blue) (Figure 2C,F,I,L). Notably, mineralization appeared to progress from the outer periphery towards the implant’s center, as demonstrated in Figure 2B,E,H,K. The bone within the scaffolds formed through an intramembranous process with no clear evidence of cartilage formation or endochondral ossification.

The relative percentages of the soft tissue, bone and scaffold are presented in Figure 3A,B, and the relative percentages of direct contact bone–scaffold and soft-tissue–scaffold are shown in Figure 3C,D. When assessing the efficacy of different materials to support new bone formation, no statistically significant difference was found (*p* > 0.05), at the periphery and the center of the defect. Notably, PCL-Bioglass exhibited a slightly higher level of bone formation in the central region compared to the other scaffolds (Figure 3B). Specifically, the percentage of area occupied by the newly formed bone, at the center, was as follows: 0.21 ± 0.58%, 1.86 ± 4.84%, 0.78 ± 1.97%, and 3.07 ± 7.58% for PCL, PCL-HA, PCL-TCP, and PCL-Bioglass, respectively. A crucial characteristic of a biomaterial designed for bone regeneration is its ability to promote osteointegration [40]. Remarkably, all the materials were well integrated with the newly formed bone at the defect’s periphery, as confirmed by both visual inspection (Figure 2A,D,G,J) and the calculated relative percentage of bone–scaffold interfaces (Figure 3C). Specifically, the relative percentages of bone–scaffold interface, at the periphery, were: 30.19 ± 11.98 for PCL, 35.03 ± 16.82% for PCL-HA, 31.22 ± 17.86 for PCL-TCP, and 29.77 ± 24.72% for PCL-Bioglass. No statistical difference (*p* > 0.05), both at the center and at the periphery, was observed (Figure 3C, D), suggesting that all the PCL-based materials supported excellent osteointegration. In all scaffolds, bone formation at the periphery was 21.22 ± 10.87% for PCL, 19.22 ± 10.46% for PCL-HA, 24.51 ± 10.58% for PCL-TCP, and 19.87 ± 13.98% for PCL-Bioglass. This experiment lasted for 6 weeks and most probably further bone formation within the center of the scaffolds would have been observed had the experiment been for a longer period.

### 3.2. Backscattered Electron Microscopy

**Figure 4:**
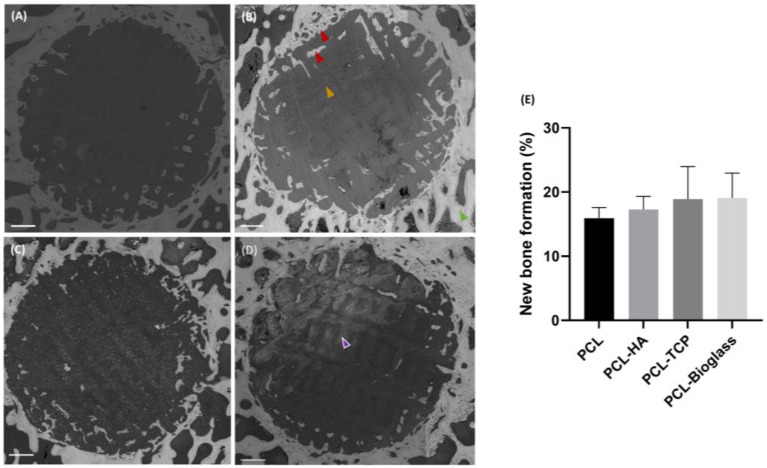
BSE images of bone cross-sections after six weeks of implantation. In panel (**A**) PCL, (**B**), PCL-HA, (**C**) PCL-TCP, and (**D**) PCL-Bioglass are displayed. The yellow arrow points to the bone brick, the green arrow indicates old bone, and the red arrows indicate new bone. The purple arrow (**D**) points to the network of PCL-Bioglass scaffold. The scale bar is set at 1 mm. (**E**) BSE analysis of % area of new bone formation in the defects is shown. Results are reported as mean ± SD (*n* = 3). One-way ANOVA was performed among different samples. The *p*-value is >0.05.

To quantify the percentage of mineralized tissue within the defect, BSE images of the entire ROI were captured. BSE is a technique that provides information about the sample’s composition by collecting scattered electrons. Areas with higher atomic numbers exhibit enhanced brightness. As shown in Figure 4, PCL-only scaffolds (consisting of carbon, hydrogen, and oxygen) appeared dark. Conversely, materials augmented with HA, β-TCP, and Bioglass (made of calcium, phosphorus, and silicon) were brighter. For instance, in Figure 4D, the distinctive network of PCL containing Bioglass particles is clearly visible (indicated by the purple arrow) although the signal was less intense compared to mineralized bone tissue. Image analysis confirmed that all biomaterials were well-integrated with the newly formed bone (lighter grey shade), and the formation of nascent bone tissue occurred within the biomaterial network. Subsequent statistical analysis of BSE images revealed no significant difference in the percentage of newly formed bone among samples (*p* > 0.05). The amounts of newly formed bone were: 15.93 ± 1.66% for PCL, 17.28 ± 2.06% for PCL-HA, 18.90 ± 5.08% for PCL-TCP, and 19.08 ± 3.88 for PCL-Bioglass (Figure 4E).

### 3.3. X-ray Computed Tomography

**Figure 5:**
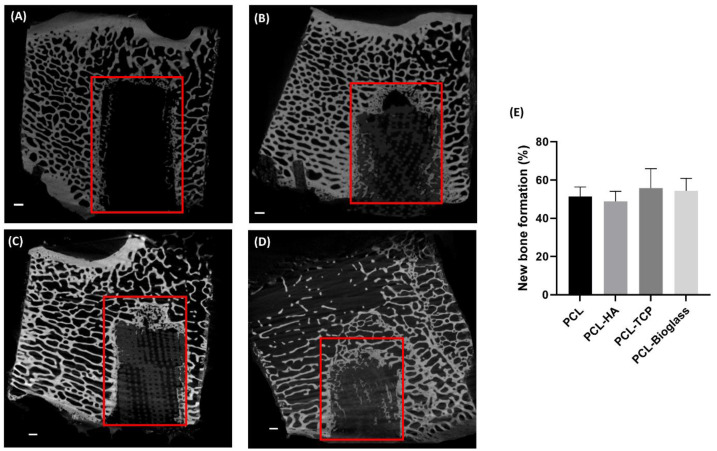
2D cross-sections of the implants using XCT imaging; (**A**): PCL, (**B**) PCL-HA, (**C**) PCL-TCP, and (**D**) PCL-Bioglass. The red square indicates the region of interest (ROI) for the segmentation of newly formed bone. The scale bars are set at 1 mm. (**E**) Percentage of new bone formation relative to the total volume. Results are reported as mean ± SD (*n* = 3). One-way ANOVA was performed among different samples. The *p*-value is >0.05.

X-ray computed tomography (XCT) imaging using Versa 520 (Carl Zeiss, Jena, Germany) was employed to image the bone structure and new bone formation throughout the entire defect (Figure 5A–D). XCT calculations provide a more accurate assessment of the mineralization process when compared to BSE, as XCT analysis is based on the total volume of the defect rather than of sections. Like BSE, the settings for XCT imaging that we used in this study were unable to differentiate between soft tissues, voids, and PCL. Consequently, the volume of the PCL plugs was estimated based on the average volume of the other three plugs (PCL-HA, PCL-TCP and PCL-Bioglass). After 6 weeks, new bone had formed at the periphery of all the defects creating a sclerotic margin around the implants. The results of the bone quantification analysis are presented in Figure 5E. No statistically significant difference was observed among the different materials (*p* > 0.05). The percentage of newly formed bone over the total volume of the defect was as follows: 51.40 ± 4.94% for PCL, 48.87 ± 5.24% for PCL-HA, 55.84 ± 10.12% for PCL-TCP and 54.34 ± 6.57% for PCL-Bioglass. The images displayed in Figure 5 depict the osteointegration of the scaffold with the periphery of the implant. The unfilled PCL implants could not be seen in the XCT; however, implants containing HA, TCP, or Bioglass were clearly detected. The defect with the PCL-Bioglass was the only one that showed bone formation in the center of the implant.

## 4. Discussion

This study, conducted over a 6-week period, investigated the osteoconduction and osteointegration of PCL-based scaffolds enriched with 20% of either HA, TCP, or Bioglass in a critical-size ovine femoral condyle defect in vivo. The scaffolds, featuring an average pore size of 330 μm, were subjected to histological and XCT analyses, revealing their potential use for bone repair and reconstruction. Notably, PCL scaffolds with 20% *w*/*w* Bioglass exhibited a slightly higher bone formation at the center of the defect compared to the other biomaterials. The analysis of XCT data indicated that the percentage of newly formed bone over the total volume of the defect ranged from 45–55%, with no statistical differences observed among the materials (*p* > 0.05). Furthermore, all biomaterials exhibited integration with the newly formed bone at the periphery of the defect, forming a tightly interlocked structure as evident from the direct contact between the biomaterials and the newly formed bone. Fuchsin-toluidine staining further revealed the presence of soft fibrous tissues within the inner pores of the scaffolds without evidence of any endochondral ossification, indicating that the bone formation primarily occurred via intramembranous ossification. Overall, these results corroborated our prior in vitro publication on hAdMSC and PCL-based scaffolds, where PCL/Bioglass exhibited the highest production of osteogenic differentiation markers (alkaline phosphatase and osteocalcin) [33].

We refer to these bone substitute materials as “bone bricks”, as they can be assembled to address large volumes of bone loss, such as defects seen in fracture, tumours and trauma [41]. Furthermore, these materials are fabricated using 3D printing technology, enabling the production of customized implants tailored to the specific requirements of the region undergoing reconstruction.

Furthermore, the bone bricks present an interconnected porous structure to support bone infiltration and regeneration in line with several other publications [42,43]. The plugs evaluated in this study were designed with a pore size of 330 µm. Theoretically, non-degradable materials should exhibit a highly interconnected pore structure, with a porosity higher than 70% and an average pore size of 300 µm, to ensure viable cell infiltration, attachment, tissue growth, and vascularization [35]. However, there is a tradeoff between strength, porosity, and stress shielding though with polymeric materials which have a lower modulus than metal alloys this last issue is less relevant.

The inclusion of bioceramics in the PCL followed from previous studies where materials without bioactive surfaces lead to comparatively reduced bone repair due the poor bone-bonding ability of scaffolds [44]. This indicates that it is advantageous to include bioactive materials in the PCL which increase strength and resilience combined with bioactivity, whereas bone graft substitute materials made solely from calcium phosphates or from Bioglasses are applied as granules and have little coherent strength. In this study, we observed that the scaffolds were not fully resorbed, and they appeared to maintain the original structure. These results are consistent with the previous literature that reported the degradation time of PCL-containing structures, as evaluated under simulated in vivo conditions, can be up to 4 years [45]. PCL is a stable polymer in aqueous media due to its high degree of crystallinity and hydrophobicity. The degradation mechanism depends on many factors related to both the material (e.g., crystallinity, molecular weight) and the architecture of the scaffold (e.g., pore size, shape). In a previous preliminary study, we performed an accelerated hydrolytic degradation study of the bone bricks (with a rectangular structure and three different pore sizes: 200, 300, and 500 µm) using sodium hydroxide (5 N) in aqueous solution. The results showed that PCL/Bioglass exhibited higher degradation kinetics than PCL, PCL/HA, and PCL/TCP. Additionally, the degradation processes increased with increasing pore size [46].

In our in vivo study, no statistical difference was observed in the amount of new mineral formation in the different scaffolds. However, the analysis of both histological and CT data revealed that the scaffold with Bioglass exhibited an improved bone formation in the center of the scaffolds. This behavior can be attributed to several factors, such as the degradation rate, the osteoconductivity, and the chemical composition of the Bioglass (SiO_2_, 24.5 wt% CaO, 24.5 wt% Na_2_O, and 6 wt% P_2_O_5_). Bioglass degrades faster than hydroxyapatite over time, releasing ions (including silicon) that are beneficial to bone formation, as they activate genes controlling the osteogenesis and growth factor production [47]. On the other hand, β-tricalcium phosphate implants degrade faster than Bioglass, leading to an imbalance between resorption and osseous regeneration, consequently ending in improper consolidation [48]. Our results align with Ghosh et al., who investigated the in vivo response of porous scaffolds of HA, β-TCP, and Bioglass implanted in radius bone defects of Bengal goats [28]. The porous struts in their study were fabricated from β-naphthalene and polyvinyl alcohol, followed by sintering, resulting in blocks of 30–35 vol% (8–160 μm). Their findings demonstrated that HA implants showed less bone in-growth and reduced mechanical anchorage compared to TCP implants, potentially due to the lower degradation of HA in the physiological environment than TCP. Few in vivo studies compare scaffolds enriched with HA, β-TCP and Bioglass45S. Przybilla et al. tested four different 20 μm coatings (HA, Bioglass, GB14 and β-TCP) on titanium implants placed in a rabbit defect model for 24 weeks, Bioglass showed the lowest fixation [29]. The authors suggested that Bioglass degraded faster than new bone formation, leading to a loss of stability (as shown by the lower shear strength after 24 weeks). On the other hand, a previous study from Moritz et al. showed that Bioglass promoted increased bone formation compared to uncoated cylindrical titanium implants, in femoral epicondyle rabbit models [49]. These conflicting outcomes may depend on the duration of the experiment, the type of implant or scaffold used and the manufacturing techniques. For instance, Moritz et al.’s study lasted only 8 weeks and the coating (obtained by CO_2_-laser) thickness was 30–40 μm.

In our study, we observed the presence of direct interaction between the surface of the implants and bone for all the materials. The phenomenon of osteointegration is a fundamental requirement for these bone substitute materials [50]. Other studies involving porous metallic materials tested in a similar ovine condylar model demonstrated very little bone growth into the inner pores unless there was a calcium phosphate coating [51]. Whist using the same model, the stiffness of the material has been shown to influence the extent of bone ingrowth into porous structures [39]. The PCL utilized in this study when combined with calcium phosphate particles or with Bioglass showed deep in-growth into the implant, resulting from the use of a low modulus material combined with bioactive particles.

Finally, this study has several limitations. Firstly, this experiment lasted for 6 weeks, and it is likely that extended experimental time, such as over 12 weeks, could reveal further bone formation within the center of the scaffolds [28,32]. This may be an important study because if bone formation was increased it would suggest that the soft tissue, we see in the defects at 6 weeks can be converted into bone through intramembranous ossification. To enhance the robustness of the results, additional controls, including untreated defects and PCL scaffolds containing HA, TCP, and Bioglass but with engineered structural stiffness above and below those used in this study, should be included in future studies. Another final limitation of this study is the use of animals from just one sex. In this study, just adult female sheep were used, which did not consider sex-dependent variability [52]. Finally, the implants were inserted into a cancellous site, but these bone bricks may also be used to treat defects in cortical diaphyseal bone where the loading environment is different from metaphyseal bone.

## 5. Conclusions

In this study, scaffolds based on PCL with an average pore size of 330 μm, containing either HA, TCP, or Bioglass were evaluated for their efficacy in promoting bone regeneration using a critical-size ovine femoral condyle defect model. All the biomaterials promoted new bone formation, 6 weeks after implantation, and all were integrated into the cancellous bone in this animal model. In all scaffolds bone formation occurred mostly at the periphery of the porous implant with less bone seen centrally.No statistical difference was found among the different biomaterials, however, PCL-Bioglass seemed to induce more bone formation in the center of the bone defect than the other types of materials. The bone within the scaffolds formed by an intramembranous process. This experiment lasted for 6 weeks, and most probably further bone formation within the center of the scaffolds would have been seen had the experiment been for a longer period. The melt blended materials can be considered a controlled degradable bone biomaterial for repair of bone tissue. Moreover, the materials showed a good biocompatibility with the tissue. These materials show great potential to promote bone regeneration, in critical-sized defects in load-bearing sites.

## Data Availability

The data presented in this study are available on request from the corresponding author.
